# Lactobacilli Regulate *Staphylococcus aureus* 161:2-Induced Pro-Inflammatory T-Cell Responses *In Vitro*

**DOI:** 10.1371/journal.pone.0077893

**Published:** 2013-10-18

**Authors:** Yeneneh Haileselassie, Maria A. Johansson, Christine L. Zimmer, Sophia Björkander, Dagbjort H. Petursdottir, Johan Dicksved, Mikael Petersson, Jan-Olov Persson, Carmen Fernandez, Stefan Roos, Ulrika Holmlund, Eva Sverremark-Ekström

**Affiliations:** 1 Department of Molecular Biosciences, the Wenner-Gren Institute, Stockholm University, Stockholm, Sweden; 2 Department of Animal Nutrition and Management, Swedish University of Agricultural Sciences, Uppsala, Sweden; 3 Department of Mathematics, Division of Mathematical Statistics, Stockholm University, Stockholm, Sweden; 4 Department of Microbiology, Swedish University of Agricultural Sciences, Uppsala, Sweden; Charité, Campus Benjamin Franklin, Germany

## Abstract

There seems to be a correlation between early gut microbiota composition and postnatal immune development. Alteration in the microbial composition early in life has been associated with immune mediated diseases, such as autoimmunity and allergy. We have previously observed associations between the presence of lactobacilli and *Staphylococcus* (S.) *aureus* in the early-life gut microbiota, cytokine responses and allergy development in children. Consistent with the objective to understand how bacteria modulate the cytokine response of intestinal epithelial cell (IEC) lines and immune cells, we exposed IEC lines (HT29, SW480) to UV-killed bacteria and/or culture supernatants (-sn) from seven *Lactobacillus* strains and three *S. aureus* strains, while peripheral blood mononuclear cells (PBMC) and cord blood mononuclear cells (CBMC) from healthy donors were stimulated by bacteria-sn or with bacteria conditioned IEC-sn. Although the overall IEC response to bacterial exposure was characterized by limited sets of cytokine and chemokine production, *S. aureus* 161:2-sn induced an inflammatory response in the IEC, characterized by CXCL1/GROα and CXCL8/IL-8 production, partly in a MyD88-dependent manner. UV-killed bacteria did not induce a response in the IEC line, and a combination of both UV-killed bacteria and the bacteria-sn had no additive effect to that of the supernatant alone. In PBMC, most of the *Lactobacillus-sn* and *S. aureus-sn* strains were able to induce a wide array of cytokines, but only *S. aureus-sn* induced the T-cell associated cytokines IL-2, IL-17 and IFN-γ, independently of IEC-produced factors, and induced up regulation of CTLA-4 expression and IL-10 production by T-regulatory cells. Notably, *S. aureus*-sn-induced T-cell production of IFN- γ and IL-17 was down regulated by the simultaneous presence of any of the different *Lactobacillus* strains, while the IEC CXCL8/IL-8 response was unaltered. Thus these studies present a possible role for lactobacilli in induction of immune cell regulation, although the mechanisms need to be further elucidated.

## Introduction

The early gut microbiota composition influences our immune system, particularly in the early post-natal period of immune development [1]. For example, germ-free (GF) mice have impaired regulatory function and induce less tolerogenic responses compared to conventional mice. Subsequent colonization of these GF mice with single bacteria species corrects immune function [2-4]. Further, the gut microbiota is also important for intestinal homeostasis and epithelial cell function [5]. Intestinal epithelial cells (IEC) express pattern recognition receptors (PRRs) and respond to bacterial stimulation by secreting cytokines, chemokines and antimicrobial peptides [6]. However, still very little is known regarding how epithelial cells influence the interaction between gut microbes and the immune cells [7]. 

The epithelial cell layer creates a barrier separating the luminal content from the underlying tissue as well as facilitating exchange of nutrients and antigens. Through enterocytes, microfold (M) cells, goblet cells and trans-epithelial dendritic cells sampling and processing of luminal antigens can occur, inducing regulatory or inflammatory immune response in intestinal T-cells [8,9]. In humans, there is an association between early microbiota composition and immune mediated diseases, such as allergy and autoimmunity [10]. Thus unfavourable microbiota composition, also termed dysbiosis, could influence the host immune homeostasis [2]. 

Traditionally, *Escherichia coli* and *Bifidobacterium* species have been considered as common early gut colonizers, but nowadays *Staphylococcus*(S.) *aureus*, a Gram-positive bacterium commonly found on the skin and on mucosa of nasal passages, is also frequently isolated from infant stools [11,12] and its presence in the gut has been associated with allergy development [13]. Strains of *S. aureus* can produce enterotoxins, capable of functioning as superantigens that activate large numbers of non-specific T-cells in the gut [14]. *In vitro* stimulation of peripheral blood mononuclear cells (PBMC) with superantigenic *S. aureus* and/or other toxins from staphylococci induces IL-17 production by T-cells [15,16], a cytokine highly associated with both allergy and autoimmunity. 

Previous studies have shown that some lactobacilli may reduce T-cell responses induced by allergens, at least *in vitro* [17]. This is interesting in relation to the reported lower prevalence of lactobacilli during the first months of life in children who later develop allergic disease [18,19]. However, how lactobacilli mediate this down regulation is not entirely known. Lactic acid produced by lactobacilli has been shown to protect epithelial cells from streptococci induced damage [20] and histamine derived from *Lactobacillus* (L.) *reuteri* has been shown to suppress TNF-α release from human monocytes by binding to H_2_ receptors [21]. However, since only certain strains of lactobacilli produce histamine additional mechanisms might be involved in lactobacilli induced immune regulation and further studies are needed.

Here we investigated how *S. aureus* and *Lactobacillus* strains influence cytokine production by IEC lines and immune cells *in vitro* and whether IEC-secreted factors influence these immune responses, as we have previously found associations between these bacteria and cytokine responses in childhood [22]. IEC lines were cultured together with *S. aureus* and *Lactobacillus* culture supernatants (-sn). Also, PBMC from healthy donors or cord blood mononuclear cells (CBMC) were stimulated with the bacteria-sn alone or with bacteria conditioned IEC-sn. The IEC-, PBMC/CBMC- and T-cell production of a wide range of cytokines and chemokines were investigated with proteome array, ELISA and flow cytometry; and the effect of lactobacilli on *S. aureus*-induced responses was further evaluated. 

## Materials and Methods

### Ethics Statement

Ethical permission for this study was obtained from the Human Ethics Committee at the Karolinska Institute, Stockholm, Sweden (Dnr 04-106/1 and Dnr: 2004-M308). Informed written consent was obtained and all samples have been used and stored as specified in the approved ethical application. All samples are coded and results from the experiments are kept separately from personal records. It will not be possible to connect published data to any individual. 

### Intestinal Epithelial Cell Lines

The human colonic epithelial carcinoma cell lines, HT-29 (HTB-38) and SW-480 (CCL228) were obtained from American Type Culture Collection (ATCC, Manassas, VA, USA). Both cell lines are surface adherent enterocyte like cells. HT-29 is a well-differentiated cell line with ultra-structural features including microvilli, microfilaments, large vacuolated mitochondria with dark granules, smooth and rough endoplasmic reticulum with free ribosomes, lipid droplets, few primary and many secondary lysosomes. SW-480, on the other hand, is simple squamous undifferentiated cells with long cellular projection and irregular sparse microvilli. (ATCC product information: www.lgcstandards.com/atcc) [23]. Both HT-29 and SW-480 are reported to express different sets of TLRs, but the level of expression varies significantly between studies [24,25].

HT-29 and SW-480 were cultured at 37°C, 5% CO_2_ in McCoy’s 5A medium modified (ATCC or HyClone Laboratories, Inc, South Logan, UT, USA) and in 1:1-mixture of DMEM (ATCC) and Leibovitz’s L-15 medium (HyClone Laboratories, Inc), respectively. Both media were supplemented with 10% heat-inactivated fetal calf serum (FCS) (Gibco, Invitrogen, Auckland, New Zealand) and 1% penicillin-streptomycin (PEST) (Thermo Scientific, Logan, UT, USA), respectively. Cells were kept in exponential growth phase and subcultured before reaching confluence, using trypsin-EDTA (Gibco, Invitrogen). Cells ranging 3-40 passages were used for the stimulation experiments described below.

### Bacterial strains

Different strains of *Lactobacillus* and *S. aureus* were used for stimulation experiments: *Lactobacillus. rhamnosus* GG (ATCC 53103; isolated from the probiotic product Culturelle), *L. rhamnosus* Kx151A1 (isolated from human stomach mucosa at the Swedish University of Agricultural Sciences, Genbank accession number GQ141811)*, L. reuteri* DSM 17938 [26] and *L. reuteri* ATCC PTA 4659 [27] (both are kind gifts from Biogaia AB, Stockholm, Sweden)*, L. casei* Shirota (isolated from the probiotic product Yakult), *L. casei* LMG 6904 (Belgian Co-ordinated Collections of Micro-organisms), *L. paracasei* F19 [28] (a kind gift from Arla Foods, Stockholm, Sweden), and *S. aureus* 139:3 (which has the gene for toxic shock syndrome toxin), 151:1 (which lacks genes for toxins) and 161:2 (which produces staphylococcal enterotoxin A and H). All *S. aureus* strains are kind gifts from Åsa Rosengren, The National Food Agency, Uppsala, Sweden, who also screened the strains for toxin genes by using PCR [29]. A previously reported histamine producing *Lactobacillus*, *L. reuteri* ATCC PTA 6475, was used as a positive control in the histamine ELISA [21]. The lactobacilli were cultured in tubes with 10 ml MRS broth (Oxoid, Hampshire, UK) at 37°C for 20 h and the staphylococci in tubes with 10 ml BHI broth (Merck, Darmstadt, Germany) at 37°C for 72 h (both in still culture). The bacteria were centrifuged at 14, 000 x g to collect the bacteria-sn, which were then sterile filtered (0.2 μm) and frozen at -20°C until used.

### Bacterial stimulation of IEC lines

HT-29 or SW-480 cells were trypsinated, washed and seeded at a concentration of 1x10^6^ cells/ml with 200 μl/well in 48 well plates (Costar, Cambridge, UK) and grown over night at 37°C, 5% CO_2_.

The IEC were washed and then stimulated for 24 hours with each bacteria-sn corresponding to 5% in complete cell culture medium. All *Lactobacillus-*sn were first diluted 1:1 with HEPES (HyClone Laboratories, Inc) to neutralize the pH. For co-stimulation experiments, IEC were stimulated with *S. aureus* strain 161:2-sn 1:1 with each *Lactobacillus* strains-sn. For IEC stimulation with UV-killed bacteria (*L. reuteri* DSM 17938 and/or *S. aureus* 161:2), 2x10^6^ UV-killed bacteria were added to the total volume of 200μl/well. As controls, IEC were kept in culture medium (with or without bacterial culture medium), or stimulated with 100 ng/ml lipopolysaccharide (LPS) (Sigma-Aldrich, Stockholm, Sweden), 1μg/ml PAM3CSK4 (InvivoGen, San Diego, CA, USA) or 1 μg/ml peptidoglycan (PGN) (Sigma-Aldrich). Thorough kinetic and titration studies were performed to determine the optimal time-points and concentrations for each experiment.

Supernatants from IEC stimulations were collected and frozen at -20°C until cytokine analyses or for stimulation of PBMC.

### Small interfering RNA transfection

Pre-engineered MyD88 targeting siRNA (SC-35986) and control siRNA (SC-37007) were purchased from Santa Cruz Biotechnology (Santa Cruz, California, USA). After performing initial dose-ranging studies, 60 pM siRNA was chosen for all knock down experiments. 50,000 HT29 cells were transfected with siRNA for 5 hours in 24 well plates in serum reduced siRNA transfection medium (SC-36868), in the presence of siRNA importer transfection reagent (SC-29528) according to the manufacturer’s instructions. The next day after transfection, cells were stimulated for 24 hours with 5% *S. aureus* 161:2-sn, 100 ng/ml LPS or culture medium. Supernatants were harvested and analysed for levels of CXCL8/IL-8 with ELISA. 

### Stimulations of PBMC and CBMC

Blood samples from healthy adult individuals were collected and PBMC were isolated by Ficoll-Hypaque (GE Healthcare Bio-Sciences AB, Uppsala, Sweden) gradient centrifugation. Cord bloods (CB) were collected from children born to mothers with full term pregnancies with uncomplicated deliveries. Cord blood samples were aspirated from the umbilical cord vein into heparinized vacutainer tubes after careful wiping of the cord with alcohol. PBMC and CBMC were isolated and cryopreserved as previously described [30]. The PBMC/CBMC were thawed and washed three times in complete culture medium (RPMI-1640 (Invitrogen) supplemented with 10% heat inactivated fetal calf serum, 1% PEST, 2% L-glutamine and 4% HEPES (HyClone Laboratories, Inc), before being seeded in flat-bottomed 96-well plates (Sarstedt Inc., Newton, NC). 

For stimulations of PBMC with bacteria-sn alone, 1x10^6^ cells/ml were stimulated with different bacteria-sn for 24 hours. For stimulations with bacteria-sn containing IEC soluble factors, supernatants from the different bacterial-IEC stimulations were added to PBMC/CMBCs for 24 hours. All stimulations corresponded to a final concentration of 2.5% of the bacteria-sn. Thorough kinetic and titration studies were performed to determine the optimal time-points and concentrations for each experiment.

For co-stimulation experiments, PBMC were stimulated with IEC-sn conditioned with *S. aureus* 161:2-sn together with each and every of the *Lactobacillus-*sn in 1:1 ratio, as described above.

As controls, cells were incubated in culture medium with or without bacterial culture medium (negative controls) or stimulated with 50 ng/ml LPS (Sigma-Aldrich) or 0.5 μg/ml PGN (Sigma-Aldrich) (positive controls). 

To investigate if histamine was involved in down modulation of the inflammatory response in PBMC induced by *S. aureus*, histamine levels in bacteria-sn were measured by ELISA as described below. Further, PBMC were pre-incubated with the H_2_ receptor blocking agent ranitidine (Sigma-Aldrich) for three hours before stimulating the cells with the bacteria-sn as described above. PBMC-sn were collected through centrifugation and stored at -20°C until cytokine analyses by ELISA as described below. 

To evaluate if the *Lactobacillus-*sn could degrade cytokines, 25 ng/ml of rIL-17 (R&D Systems Europe Ltd., Abingdon, UK) and 10 μg/ml of rIFN-γ (Mabtech AB, Nacka, Sweden) were pre-incubated in a 1:1 ratio with *L. reuteri* DSM 17938 or with dilution buffer for 2 hrs. The respective proteins levels were then measured with ELISA as described below.

### Cytokine array

Human cytokine array kit (Proteome Profiler TM Array with human cytokine array panel A, R&D Systems) was used to simultaneously assay the relative levels of 36 cytokines and chemokines in selected supernatants. Briefly, supernatants from IEC and PBMC stimulated with *L. reuteri* DSM 17938-sn and/or *S. aureus* 161:2-sn were incubated with detection antibody cocktails for one hour. Meantime, the antibodies coated nitrocellulose membranes were blocked using blocking buffer. The pre-incubated supernatants were added to the membranes and incubated on a rocking platform at 2-8 °C overnight. After washing, streptavidin-HRP was added. After 30 min incubation, the nitrocellulose membrane was exposed to ECL reagent (GE Healthcare UK Limited, Buckinghamshire, UK) for one minute to develop chemiluminescent spots. Images were captured using a luminescent image analyzer (Fujifilm, LAS-100 plus, Tokyo, Japan). In order to quickly identify the positive signals on developed image, a transparent overlay template was placed on the array image film and aligned with the pairs of reference spots in three corners of each array. Those three pairs of reference spots were also used for qualitative comparison on the density of each spot within the membrane. In addition, the spot size and integrated density (Integrated density = area of the spot x mean grey value) was determined using ImageJ software (NIH, Bethesda, MD). Only cytokines and chemokines detected in IEC-sn and PBMC-sn are included in Table 1 and Table 2, respectively.

**Table 1:** Cytokine profiling of supernatants from IEC stimulated with bacteria-sn.

**Proteins**	**HT-29**			
	***L. reuteri* DSM 17938**	***S. aureus* 161:2**	**LPS**	**Culture medium**
**IL-2**				
**IL-17**				
**IFN-γ**				
**IL-1α**				
**IL-1β**				
**IL-1 RA**	(+)	(+)	(+)	(+)
**IL-6**				
**IL-10**				
**IL-16**				
**TNF-α**				
**CXCL1/ GROα**		+	+	
**CXCL8/IL-8**	(+)	+	+	(+)
**CXCL10/IP-10**				
**CCL2 /MCP-1**				
**CCL3/MIP-1α**				
**CCL4 /MIP-1β**				
**CCL5/RANTES**				
**MIF**	+	+	+	+
**G-CSF**				
**GM-CSF**				
**PAI-1/SerpinE1**	(+)	(+)	(+)	(+)
**C5a**				

(+) indicates a spot with lower density than reference spots.

^+^ signifies a spot with similar or greater density than reference spots

Blank signifies undetected.

Reference spots refer to the control spots within the array.

**Table 2:** Cytokine profiling of supernatants from PBMC stimulated with bacteria-sn or bacteria conditioned IEC-sn.

**Proteins**	**Stimulation with bacteria-sn**				**Stimulation with bacteria conditioned IEC-sn**			
	***L. reuteri* DSM 17938**	***S. aureus* 161:2**	**LPS**	**Culture medium**	***L. reuteri* DSM 17938**	***S. aureus* 161:2**	**LPS**	**IEC**
**IL-2**		(+)				(+)		
**IL-17**		(+)				(+)		
**IFN-γ**		(+)				(+)		
**IL-1α**	+	+	+	(+)	+	+	(+)	(+)
**IL-1β**	+	+	(+)	(+)	+	+	(+)	(+)
**IL-1 RA**	+	+	+	+	+	+	+	+
**IL-6**	+	+	+	(+)	+	+	+	+
**IL-10**	(+)	(+)	(+)		(+)	(+)		
**IL-16**	(+)	(+)	(+)	(+)	(+)	(+)	(+)	(+)
**TNF-α**	(+)	+	+		(+)	(+)		
**CXCL1/GROα**	+	+	+	+	+	+	+	+
**CXCL8/IL-8**	+	+	+	+	+	+	+	+
**CXCL10/IP-10**		(+)		(+)		(+)		(+)
**CCL2/MCP-1**	+	+	+	+	+	+	+	+
**CCL3/MIP-1α**	+	+	+	+	+	+	(+)	(+)
**CCL4/MIP-1β**	(+)	+	+		+	+	(+)	(+)
**CCL5/RANTES**	+	+	(+)	(+)	+	+	(+)	(+)
**MIF**	+	+	+	+	+	+	+	+
**G-CSF**	+	+	+		+	+		
**GM-CSF**	(+)	(+)	(+)		(+)	(+)		
**PAI-1/SerpinE1**	(+)	(+)	(+)	(+)		(+)	(+)	(+)
**C5a**	(+)^c^	(+)	(+)	(+)	(+)	+	(+)	(+)

(+) indicates a spot with lower density than control spots

+ signifies a spot with similar or greater density than control spots.

Blank signifies undetected.

### ELISA

For ELISA determinations, commercially available kits for CXCL8/IL-8, TSLP (thymic stromal lymphopoietin), APRIL (a proliferation inducing ligand), TGF-β1, IL-6, IL-10, IL-17, IL-22 (DuoSet ELISA, R&D Systems), IFN-γ, TNF-α (Mabtech AB), histamine (IBL international GMBH, Hamburg, Germany) and IDO (indoleamine 2,3-dioxygenase) (TSZ ELISA, Framingham, MA, USA) were used according to the instructions from the manufacturers. The optical density was determined using a micro-plate reader (Molecular Devices Corp, Sunnyvale, CA, USA) set at 450 nm or 405 nm, accordingly. Results were analyzed using SoftMax Pro 5.2 rev C (Molecular Devices Corp). 

### FACS analysis of cytokine-producing T-helper cells and T-regulatory cells

PBMC were stained for surface and intracellular markers using the following antibodies: *T-helper cells*: CD4 PerCP-Cy5.5 (clone: SK3), IL-17A PE (Clone: N49-653), and IFN-γ FITC (clone: B27) and *T-regulatory cells*: CD4 FITC (clone: RPA-T4), CD25 APC-H7 (clone: M-A251), CD127 PerCP-Cy5.5 (clone: HIL-7R-M21), FoxP3 PE (clone: 259D/C7), IL-10 APC (clone: JES3-9D7) and CTLA-4/CD152 BV421 (clone: BNI3) (all from BD Biosciences Pharmingen, San Jose, CA, USA). Cells were harvested and stained according to standard procedures for surface antigens. For intracellular cytokine detection, cells were fixed and permeabilized prior to staining. For T-regulatory cells the Transcription factor buffer set was used according to recommendations from the manufacturer (BD Biosciences). Gating was performed on the basis of forward and side scatter properties for lymphocytes followed by gating of live CD4^+^ cells using either 7AAD-binding (BD Via-Probe 7) or the LIVE/DEAD Fixable Dead Cell Stain Kit-Aqua (Invitrogen). Cytokine production was evaluated in CD4^+^ cells and CD25^high^CD127^low^FoxP3^+^ cells were analysed for intracellular IL-10 and CTLA-4 and considered as T-regulatory cells. Cells were acquired using either a FACSCalibur or a FACSVerse flow cytometer (BD Biosciences). Data were analysed with the FlowJo Software (TreeStar, Ashland, OR, USA). 

### Statistics

The statistical analyses were performed at the Division of Mathematical Statistics, Stockholm University. In order to assess differences in cytokine production between IEC stimulated with different bacteria-sn and control (culture medium), log-transformed data were analysed with a two-factor ANOVA followed by pairwise comparisons obtaining p-values and confidence intervals. Bonferroni correction was used to avoid false positives. If difference between stimulations was significant, pair wise comparison was used. Similar method was used to compare PBMC stimulated with *S. aureus* 161:2 alone, and with *S. aureus* 161:2 and *Lactobacillus* strains together.

For the data presented on PBMC stimulation with each bacteria-sn, log-transformed data was analysed using paired t-test to investigate differences in cytokine production between PBMC stimulated with different bacteria-sn and control (culture medium). Correction for multiple tests was not performed.

## Results

### Soluble factor(s) from *S. aureus* 161:2 induce CXCL8/IL-8 production by IEC in a MyD88-dependent manner

**Figure 1:**
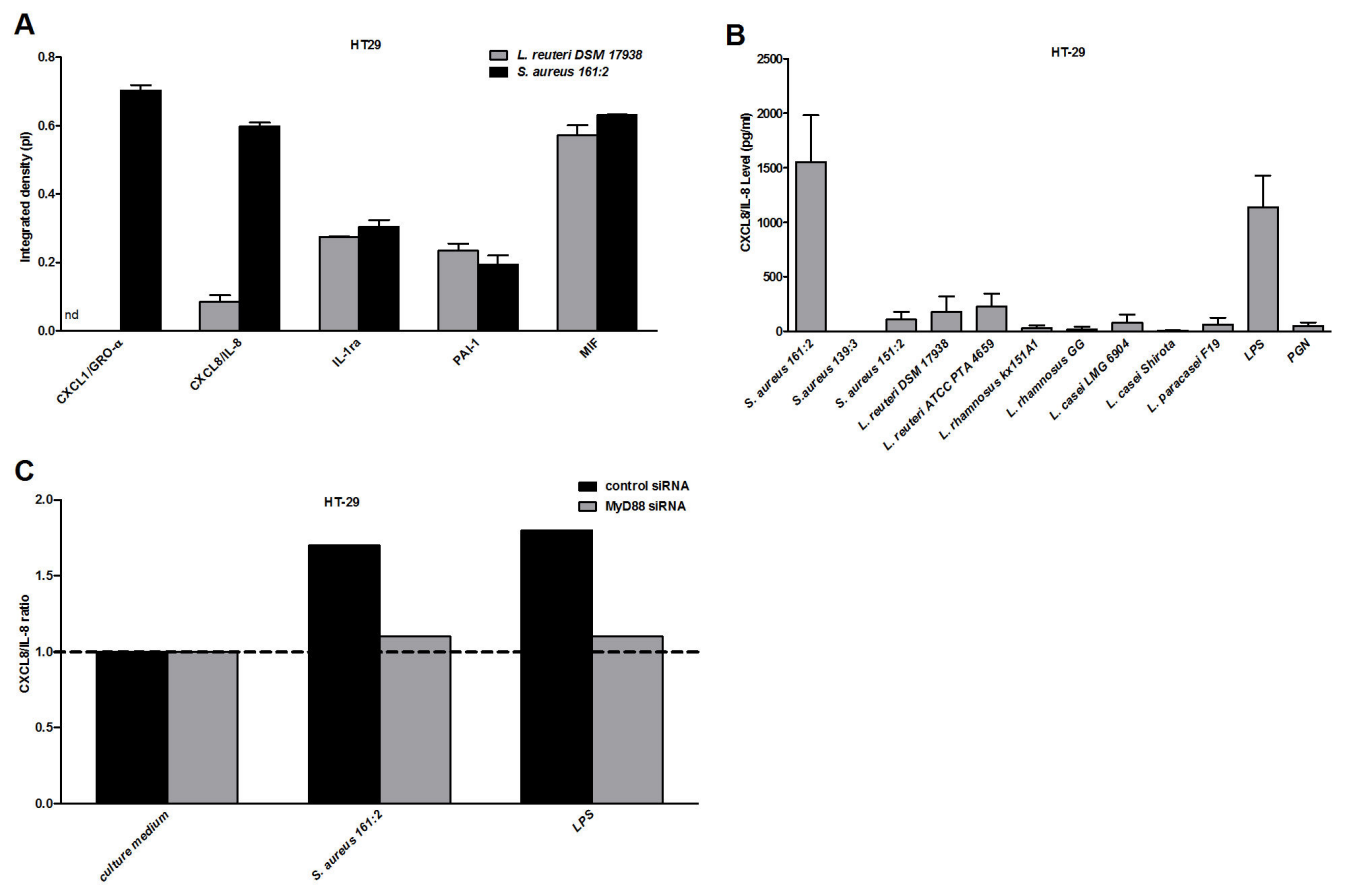
Cytokine production by IEC stimulated with bacteria supernatant. HT29 cells were cultured in 48 well plates for 24 hours with the bacteria-sn. (A) Shows semi quantitative analysis of human cytokine array in IEC-sn by measuring the integrated pixel density (Pi) (area x mean gray value). (B) Shows the level of CXCL8/IL-8 in IEC-sn following ELISA. Data are shown as means + SEM of 3-4 experiments respectively. (C) Shows the effect of MyD88-silencing on IEC CXCL8/IL-8 production normalized with the control culture medium response. One representative experiment out of three is shown. ***P < 0·001, **P < 0·01 and *P < 0·05. Background generated by bacterial medium is subtracted.

First, we screened the response of the IEC lines HT-29 and SW-480 following stimulation with soluble factors (bacteria-sn) from two bacteria (*L. reuteri* DSM 17938 and *S. aureus* 161:2) using the human cytokine Proteome Profiler TM array. The production of 36 different cytokines and chemokines was analyzed. Both IEC lines produced a restricted pattern of factors upon stimulation, but only *S. aureus* induced the production of the pro-inflammatory chemokines CXCL8/IL-8 and CXCL1/GROα above background levels (both IEC lines showed similar patterns, results are shown for HT-29 in Table 1 and Figure 1A). To confirm the finding that *S. aureus*-sn but not *Lactobacillus*-sn induce a pro-inflammatory response in IEC, we stimulated HT-29 with seven different strains of *Lactobacillus* (*L. rhamnosus* GG, *L. rhamnosus* Kx151A1*, L. reuteri* DSM 17938*, L. reuteri* ATCC PTA 4659*, L. paracasei* F19*, L. casei* Shirota and *L. casei* LMG 6904) and three *S. aureus* strains (*S. aureus* 139:3, 151:1 and 161:2) and measured the production of CXCL8/IL-8 by ELISA. Only *S. aureus* 161:2 induced IEC to produce CXCL8/IL-8 that significantly (p<0.001) differed from the background (Figure 1B). 

As IEC lines previously have been reported to produce TSLP, APRIL and TGF-β1 (cytokines implicated in epithelial-immune interactions in the gut but not included in the Proteome Array), we also investigated the production of these cytokines with ELISA. None of the bacteria-sn tested induced IEC production of any of these factors (data not shown). 

To elucidate if UV-killed bacteria could further increase the pro-inflammatory response of IEC induced by *S. aureus*-sn, we stimulated our cell lines with UV-killed bacteria, bacteria-sn or a combination of both. None of the UV-killed bacteria induced a response in the IEC line, and a combination of both UV-killed bacteria and the bacteria-sn showed no additive effect compared to the effect of the supernatant alone (Figure S1). UV-killed bacteria were therefore not included in further experiments. 

TLR-2 is involved in *S. aureus* lipoprotein recognition [31]. As IEC lines express TLRs [24], we investigated the TLR involvement in the IEC response to *S. aureus* by MyD88 silencing of the IEC. Indeed, following stimulation with *S. aureus* 161:2-sn, the IEC CXCL8/IL-8 production was reduced by the treatment (Figure 1C). 

### *S. aureus* induces an inflammatory immune response

To investigate how different bacteria influence cytokine responses by immune cells, and whether IEC-secreted factors could influence these responses, PBMC were stimulated with *L. reuteri* DSM 17938-sn and *S. aureus* 161:2*-*sn directly or with supernatants from HT-29 cultures exposed to the same bacteria. A cytokine array analysis revealed that both strains induced the production of a panel of cytokines in PBMC (including IL-6), but only *S. aureus* 161:2-sn induced the production of the T-cell associated cytokines IL-2, IL-17 and IFN-γ. The addition of IEC-derived factors did not change the cytokine profile of the PBMC (Table 2).

**Figure 2:**
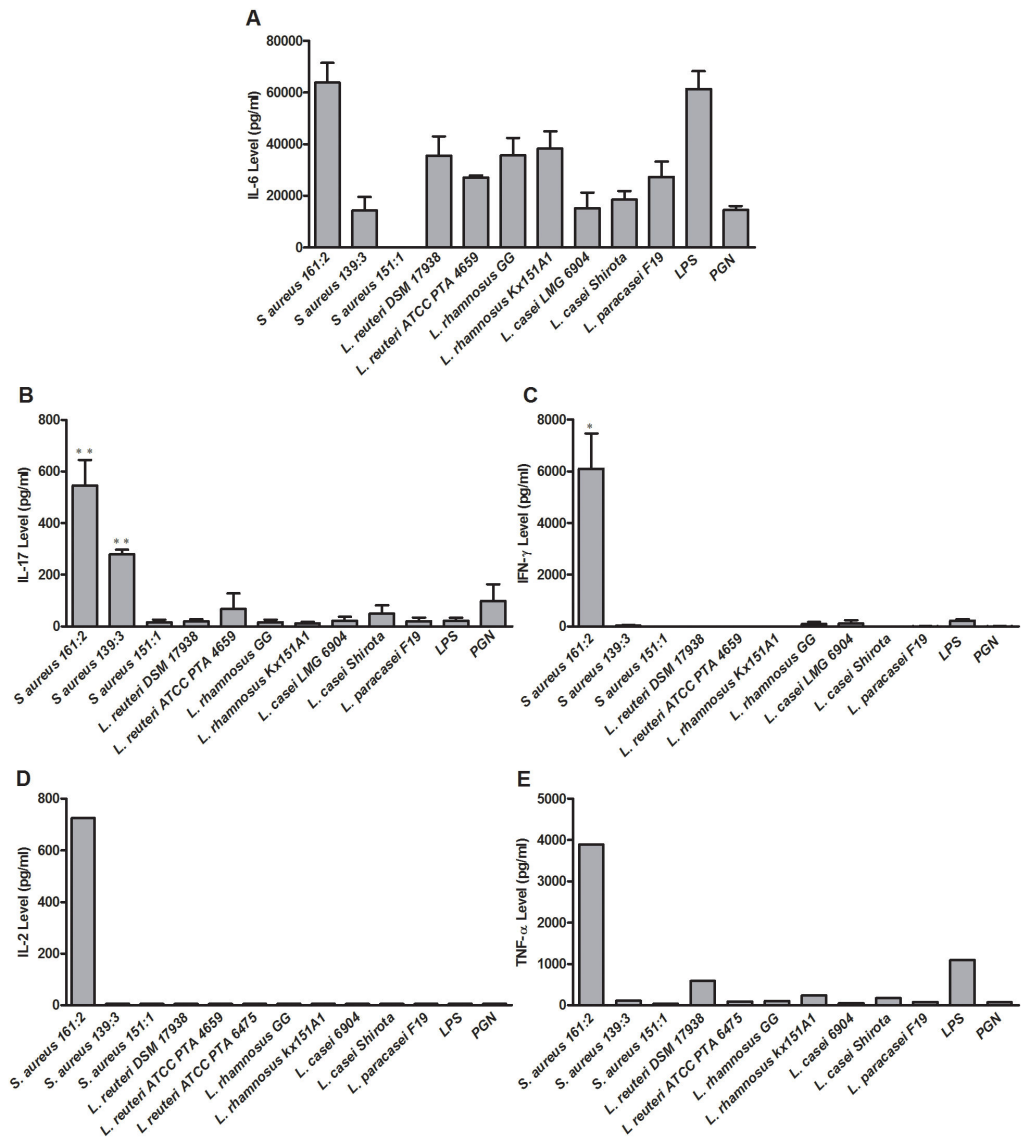
Cytokine production by PBMC stimulated with bacteria conditioned IEC-sn (A) IL-6, (B) IL-17, (C) IFN-γ, (D) IL-2 and (E) TNF-α levels measured by ELISA in supernatant from PBMC stimulated with bacteria conditioned IEC-sn for 24 hours. (A-C) The graphs represent mean + SEM value of independent experiments using PBMC from 3 healthy donors. ***P < 0·001, **P < 0·01 and *P < 0·05. (D-E) One representative experiment is shown. Background generated by bacterial medium is subtracted.

To quantify cytokines detected with the cytokine array, we investigated the ability of all bacterial strains included in this study to induce PBMC production of IL-6, IL-17, IFN-γ, IL-2 and TNF-α and measured the release with ELISA. Both *S. aureus* 161:2 and the lactobacilli were capable of inducing IL-6 production by PBMC (Figure 2A). However, only *S. aureus* induced IL-17 (*S. aureus* 161:2 and 139:3), IFN-γ, IL-2 and TNF-α production (*S. aureus* 161:2), while the lactobacilli induced none or low levels of these cytokines (Figure 2B-E). 

### Lactobacilli can down-regulate the inflammatory immune response induced by *S. aureus* 161:2

**Figure 3:**
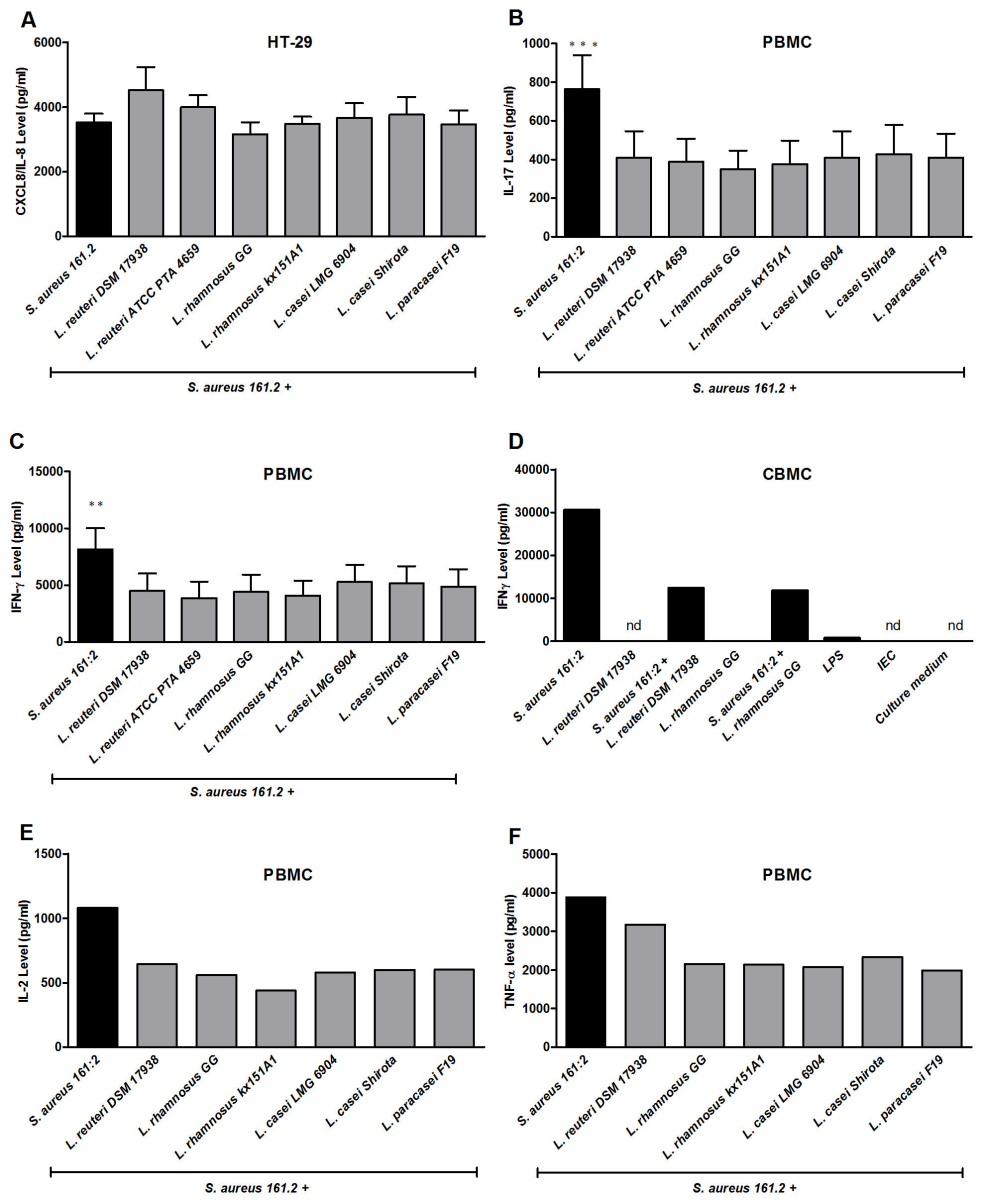
Cytokine production by intestinal epithelial cells and immune cells simultaneously co-cultured with *S. aureus* 161:2 and each *Lactobacillus* strain supernatant. (A) CXCL8/IL-8 level in IEC-sn after co-cultured with *S. aureus* 161:2-sn together with each *Lactobacillus* strains-sn measured by ELISA. (B) IL-17 and (C) IFN-γ levels in PBMC-sn after PBMC co-cultured with *S. aureus* 161:2-sn together with each *Lactobacillus* strains-sn measured by ELISA. The graphs represent mean + SEM value of independent experiments using PBMC from 3 healthy donors. (D) Shows IFN-γ production by CBMC simultaneously co-cultured with *S. aureus* 161:2-sn and either *L. reuteri* DSM 17938-sn or *L. rhamnosus* GG culture–sn. One representative CB donor out of four. nd signifies below detection. (E) IL-2 and (F) TNF-α levels in PBMC-sn after PBMC co-cultured with *S. aureus* 161:2-sn together with each *Lactobacillus* strains-sn measured by ELISA. One representative experiment is shown. ***P < 0·001, **P < 0·01 and *P < 0·05. Background generated by bacterial medium is subtracted.

Lactobacilli are reported to exert immune regulatory effects [32,33]. To investigate whether the secreted products from lactobacilli could modify the pro-inflammatory IEC and/or PBMC responses elicited by *S. aureus* 161:2, we stimulated the cells with a combination of *S. aureus* 161:2*-*sn and the different *Lactobacillus*-sn. While none of the *Lactobacillus*-sn modified the *S. aureus* 161:2 CXCL8/IL-8 response by IEC (Figure 3A), the *S. aureus* induced IL-17, IFN-γ, IL-2 and TNF-α production by PBMC was significantly reduced by a simultaneous exposure to *Lactobacillus*-secreted factors (Figure 3B-C and E-F). This was also true for CBMC, where the *S. aureus* induced IFN-γ response was reduced by the tested lactobacilli (Figure 3D). 

**Figure 4:**
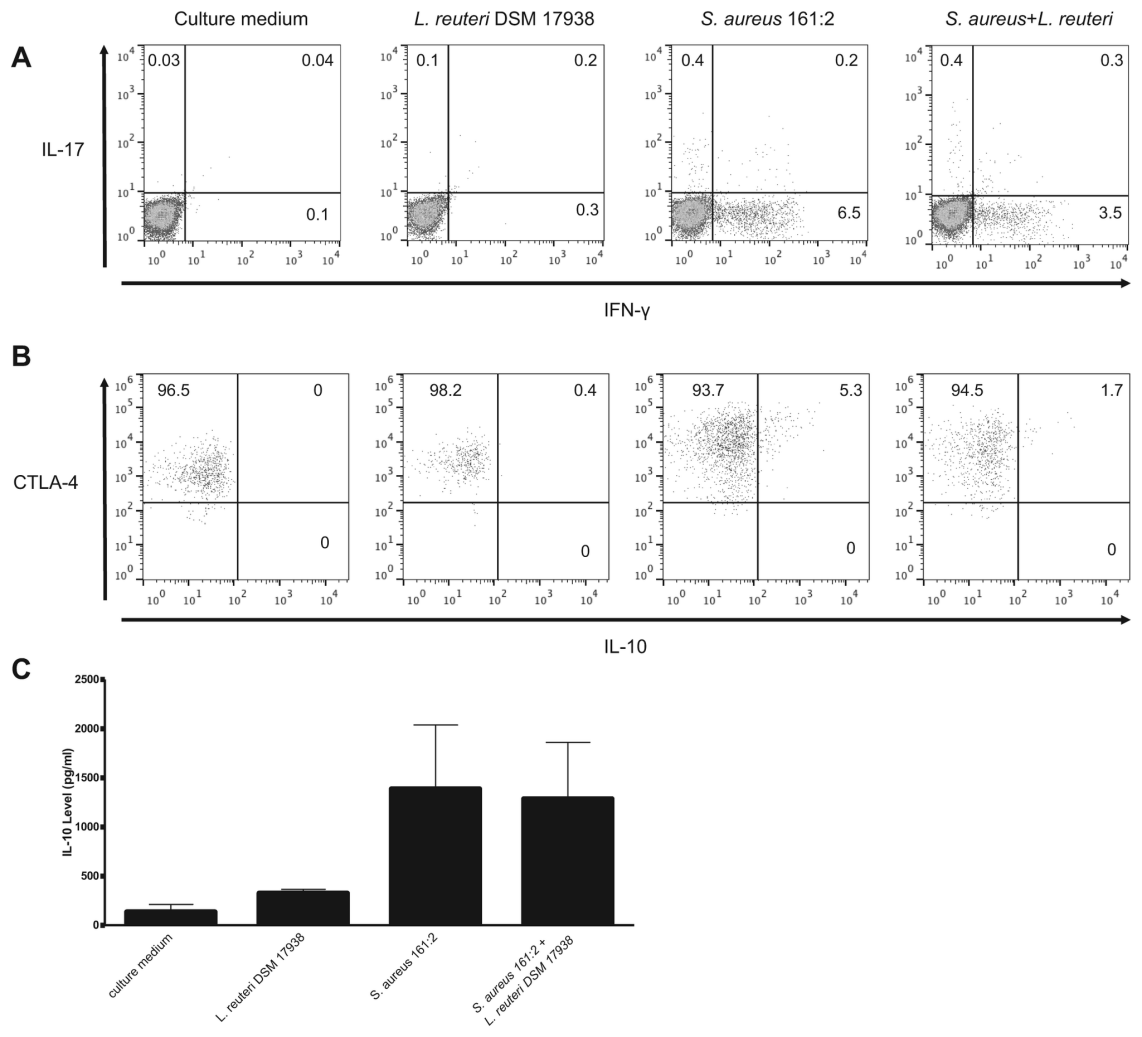
*S. aureus*-sn induces IL-17 and IFN-γ producing T-helper cells and up-regulation of CTLA-4 and IL-10 production by T-regulatory cells after *in* *vitro* stimulation of PBMC. Flow cytometry analysis of (A) IL-17^+^ and IFN-γ^+^ CD4^+^ T-cells and (B) CTLA-4^+^ and IL-10^+^ T-regulatory cells following stimulation with *S. aureus* 161:2-sn, *L. reuteri* DSM 17938-sn or a combination of both. Numbers refer to percentages of positive cells. One representative experiment out of five, using healthy adult donors. (C) Shows IL-10 production by PBMC stimulated with *S. aureus* 161:2-sn, *L. reuteri* DSM 17938-sn or a combination of both. The graphs represent mean + SEM value of independent experiments using PBMC from 3 healthy donors.

The IFN-γ and IL-17 production following *S. aureus* stimulation was attributed to T-cells as shown by analysis of the stimulation experiments by flow cytometry (Figure 4A). Further, we observed that a simultaneous stimulation with *L. reuteri* DSM 17938-sn and *S. aureus* 161:2-sn decreased the percentage of IFN-γ secreting cells (Figure 4A). In addition, *S. aureus* affected the T-regulatory cell population by up regulating CTLA-4 expression (culture medium GeoMFI 1269, *L. reuteri* DSM 17938 GeoMFI 2669, *S. aureus* 161:2 GeoMFI 8259,) and inducing production of IL-10, while the simultaneous stimulation with *L. reuteri* DSM 17938-sn slightly dampened the CTLA-4 expression (*S. aureus* 161:2+*L. reuteri* DSM 17938 GeoMFI 4606) and the IL-10 response (Figure 4B-C).

### Studies on lactobacilli mediated regulation of *S. aureus*-induced responses

**Figure 5:**
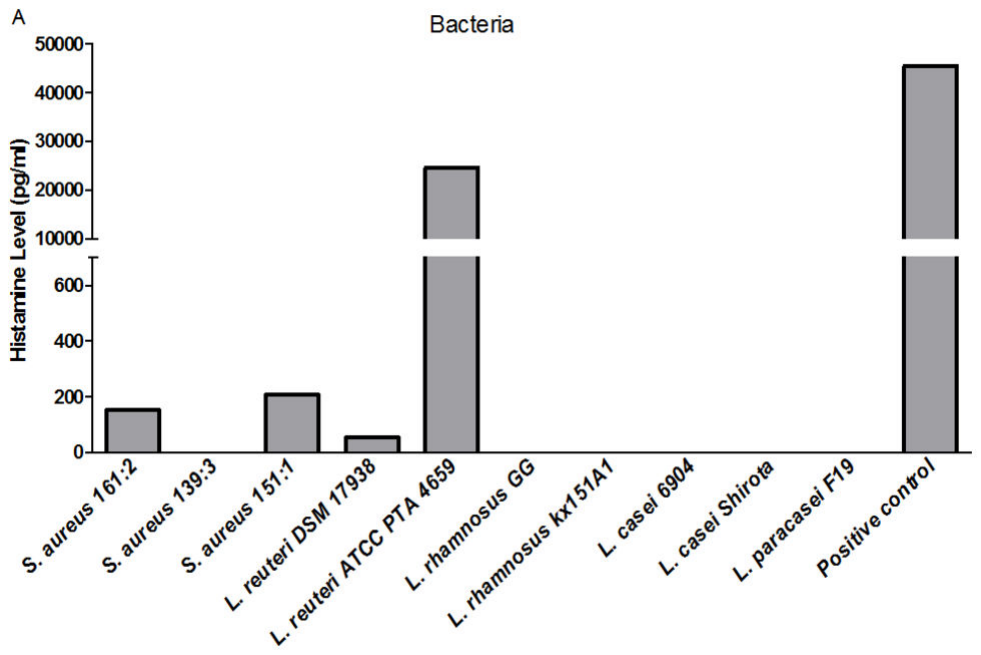
*Lactobacillus* strains differentially produce histamine. ELISA analysis of histamine level in the bacteria supernatants. Histamine production by *L. reuteri* ATCC PTA 6475 was used as a positive control. One representative experiment out of two is shown.

The enzyme IDO can be induced by commensal bacteria and has been shown to favor the induction of FoxP3^+^ T-regulatory cells [34] and to suppress Th17-cell development [35]. To test the hypothesis that lactobacilli induce IDO production by immune cells, we measured the level of IDO in the PBMC supernatant stimulated with different bacteria-sn conditioned IEC-sn. However, none of the bacteria induced IDO above background levels (data not shown). Histamine derived from some lactobacilli has been shown to down-regulate inflammatory immune responses in human monocytes by binding to H_2_ receptors [21]. To investigate if histamine derived from lactobacilli could be responsible for the down modulation of the *S. aureus* 161:2 induced inflammatory response, we examined histamine production from our different strains of bacteria. However, only one out of our original set of seven lactobacilli (*L. reuteri* ATCC PTA 4659) produced histamine (Figure 5). To further verify that histamine did not play a role in the regulation of *S. aureus*-induced immune responses in our experimental system, we also blocked H_2_ receptors and subsequently stimulated PBMC with a combination of *S. aureus* 161:2-sn and *L. reuteri* DSM 17938-sn. No effect on the lactobacilli-mediated regulation was observed (data not shown). 

## Discussion

Today, many neonates are colonized with *S. aureus* in the intestine and its presence in the gut early in life has been associated with allergy development [13]. In contrast, the colonization with lactic acid-producing bacteria seems to decrease in westernized countries, and their reduced prevalence is associated with the development of allergies [18,19]. We have recently demonstrated strong relations between the early colonization pattern of *S. aureus* and lactobacilli and cytokine production at two years of age [22] indicating that these bacteria can have impact on immune responses during childhood and potentially influence the onset of immune-mediated diseases like allergy. 

Here we investigated how lactobacilli and/or *S. aureus* influence cytokine production by IEC lines as well as PBMC *in vitro*. A wide analysis of the response to *S. aureus* 161:2-sn and *L. reuteri* DSM 17938-sn by proteome array (including 36 different cytokines and chemokines) together with additional ELISA measurements of APRIL, TSLP and TGF-β1, revealed a restricted response of the IEC lines to secreted factors from *S. aureus*, characterized by CXCL8/IL-8 and CXCL1/GROα production (Table 1 and Figure 1A). A more quantitative and species-specific analysis of the induced CXCL8/IL-8 production from the IEC lines by ELISA confirmed that only culture supernatants from the enterotoxin A and H producing *S. aureus* 161:2 was able to induce CXCL8/IL-8 production by the IEC (Figure 1B). The *S. aureus*-induced IEC response was mediated by secreted bacteria products and not by the UV-killed bacteria, in agreement with previous work [36].

There is a debate on whether IEC develop a hypo-responsiveness to bacterial stimuli [37] or play an active role in priming immune cells to a tolerogenic phenotype by the secretion of TSLP and TGF-β [38]. The IEC lines used in our study do express TLRs, albeit at low levels [24,25] and our experiments showed that MyD88-silencing partially dampened the *S. aureus*-induced IEC response. Still, as very few factors were actually produced upon bacterial contact (Table 1) and the complete absence of tolerogenic mediators like TSLP and APRIL in our cultures, the results from our study rather support the idea of IEC hypo-responsiveness to bacterial stimulation. In addition, IEC- produced factors had a minor influence on the PBMC response to bacteria. Although this could result from the fact that we used non-polarized IEC (HT-29) in our experiments, we have seen similar types of responses to *S. aureus*- and lactobacilli-sn in polarized adult IEC (Caco-2) (unpublished data), supporting our findings reported here. Further, also the fetal IEC cell line (FHS-74 int) responded to the bacterial-sn in a comparable way, suggesting that *S. aureus*-sn was able to stimulate IEC lines regardless of degree of maturity. However, this does not rule out a modulatory role of the intestinal epithelium; it might rather reflect the fact that we have an *in vitro* system with IEC lines and not primary cells. In addition, the stimulatory effect of live whole bacteria might differ from secreted bacterial components. However, UV-killed bacteria did not evoke a response on its own and the addition of UV-killed bacteria to the cultures stimulated with bacterial supernatant did not increase the response (Figure S1). 

When investigating cytokine responses by PBMC following exposure to bacteria-sn with proteome array, both lactobacilli and *S. aureus* were capable of inducing many different cytokines, but only *S. aureus* 161:2 induced the production of the T-cell associated cytokines IL-2, IL-17 and IFN-γ (Table 2 and Figure 2). Flow cytometry analysis of stimulated PBMC confirmed the T-cell involvement, as IL-17 and IFN-γ secreting Th-cells were detected upon stimulation with *S. aureus* 161:2 (Figure 4A), although the number of IL-17-positive cells was very low, which is in agreement with previous findings [16]. Still IL-17 was readily detected in the supernatants of *S. aureus*-stimulated PBMC. It should be noted, that we only investigated the CD4^+^ T-cell population in this study and did not further characterize the nature of our *S. aureus*-responding T-cells; therefore we cannot exclude the contribution of CD8^+^ T-cells or unconventional T-cells to the secreted cytokines detected in the PBMC-sn. Indeed, γδ T-cells are described to express different TLRs and respond directly (as well as indirectly via dendritic cells) to bacterial products with both IL-17 and IFN-γ production [39]. 

Interestingly, *S. aureus* 161:2 also induced changes in the T-regulatory cell population by increasing CTLA-4 expression and inducing IL-10 production by these cells, suggesting that *S. aureus* also induces a simultaneous regulatory response (Figure 4B-C). 

By what mechanism(s) *S. aureus* induced cytokine production by T-cells in our system remains to be further investigated. It could be due to its production of toxins, as staphylococcal toxins can act as superantigens and cause a non-specific activation of T-cells by direct cross-linking of the T-cell receptor. Still, in our system, only one out of two superantigenic *S. aureus* induced strong T-cell associated responses with IL-17, IFN-γ, IL-2 and TNF-α production, indicating that other routes of activation may operate as well. Further, our observation of a MyD88 involvement in the CXCL8/IL-8 production by IEC line following staphylococcal exposure also suggests that *S. aureus* may activate cells in other (TLR-dependent) ways. It was recently demonstrated that lipoproteins from *S. aureus* induce T-cell activation in a TLR2-dependent way [40]. Further, ATP from e. g. *S. aureus* has been linked to Th17 induction in the gut [41], although we could not prove ATP to be a major contributing factor to *S. aureus*-induced IL-17 production in our experimental setup (unpublished observations). 

Both IL-17 and IFN-γ are associated with intestinal inflammation [42] and their induction in the gut would need to be tightly regulated to maintain epithelial integrity and function as well as immune balance. Lactobacilli are reported to support epithelial homeostasis, reduce intestinal inflammation as well as exert immune regulatory effects in general [43]. Lactobacilli can block *S. aureus* expansion in the gut by interfering with the adhesion of bacteria to the mucosal surface [44], and also modulate the immune response against *S. aureus* toxins *in vitro* [17], but the exact mechanism behind these effects remain elusive. None of the *Lactobacillus*-sn were able to down-regulate the CXCL8/IL-8 production induced by *S. aureus*, further suggesting that the epithelial responsiveness to bacterial stimulation might be limited (Figure 3A). In contrast, the simultaneous presence of *Lactobacillus*-sn and *S. aureus*-sn reduced the PBMC production of the T-cell-associated cytokines IL-17 and IFN-γ with 30-50% (Figure 3B-C) as well as IFN-γ producing cells (Figure 4A). Lactobacilli were also able to regulate the response of immature immune cells, as soluble factors from lactobacilli readily suppressed the *S. aureus*-sn induced IFN-γ production by CBMC (Figure 3D). 

Lactobacilli are reported to selectively induce regulatory immune responses and particularly IL-10 production [45]. However, we did not observe a strong IL-10 response following exposure to lactobacilli; rather the strongest IL-10 response was elicited by *S. aureus* 161:2 (Figure 4B-C). Also, while *S. aureus* induced a notable increase in the number of IL-10^+^ T-regulatory cells as well as the up-regulation of CTLA-4 in these cells, this was not seen following exposure to lactobacilli, and the simultaneous presence of *S. aureus* and lactobacilli slightly dampened T-regulatory cell induction. 

We also investigated whether IDO, an enzyme which supports the generation of T-regulatory cells and suppresses Th17 cells [34,35], were involved in the regulation of *S. aureus*-induced Th17 responses. However, none of the lactobacilli strains induced IDO above background levels, making IDO an unlikely candidate for the reduced Th17 response seen after lactobacilli-*S. aureus* co-culture.

Histamine secreted from *L. reuteri* ATCC PTA 6475 has been shown to abrogate TNF-α production by monocytes [21]. Only one of our original seven *Lactobacillus* strains (*L. reuteri* ATCC PTA 4659) was found to produce significant histamine levels (Figure 5). Further when we blocked the action of histamine (e.g. blocking H_2_ receptors) this did not alter the lactobacilli-mediated reduction of *S. aureus*-induced responses indicating that other mechanisms are involved. 

A previous *in vitro* study has revealed that *Lactobacillus* strains use lactic acid to inhibit cytotoxic inflammation induced by lipoteichoic acid, an active inflammation-inducer produced by streptococci [20]. In this study, the *Lactobacillus*-sn were treated with HEPES to neutralize the pH, excluding low pH as an explanation for the observed effect. Further, pre-incubation of recombinant IL-17 and IFN-γ with *Lactobacillus*-sn did not alter their detection by specific ELISA (our unpublished observations). However if the lactic acid could contribute to down modulation of the inflammatory responses induced by *S. aureus* 161:2 in other ways, needs further investigation. Finally, the dampening effect mediated by lactobacilli was not due to killing of the cells, as the *Lactobacillus*-sn did not influence T-cell viability (data not shown). 

The recent rise in inflammatory diseases in western countries has partly been attributed to alterations in gut microbiota. Results from this study provide insight to mechanisms behind gut microbial influence on host immune homeostasis. As indicated here, *S. aureus* induces a strong pro-inflammatory response by IEC and a prominent Th1 and Th17 response by immune cells, while lactobacilli modulate the Th1/Th17 response by immune cells. This is in agreement with our previous findings that *S. aureus* is a frequent colonizer early in life in general, while lactobacilli are less frequent early in life in children developing allergy [18], and that there is a strong correlation between the absence of lactobacilli and elevated cytokine responses during childhood [22]. Our results from this study further suggest that immuno-modulatory signals from lactobacilli are needed to direct the immune response away from an allergic/inflammatory phenotype.

## Supporting Information

Figure S1 **Cytokine production by IEC stimulated with bacteria (supernatant or UV-killed).** Level of CXCL8/IL-8 in HT29 supernatant collected after co-cultured with *Lactobacillus* and *Staphylococcus* strain-sn and/or Ultraviolet-killed (UV) bacteria, respectively. The graph represent mean + SEM value of 3 independent experiments. Background generated by bacterial medium is subtracted.
(TIF)
