# Supplementary figures and images for: Lactobacilli Regulate Staphylococcus aureus 161:2-Induced Pro-Inflammatory T-Cell Responses In Vitro
Source: PLoS One. 2013 Oct 18;8(10):e77893. doi: 10.1371/journal.pone.0077893 (PMC3799733; doi:10.1371/journal.pone.0077893)

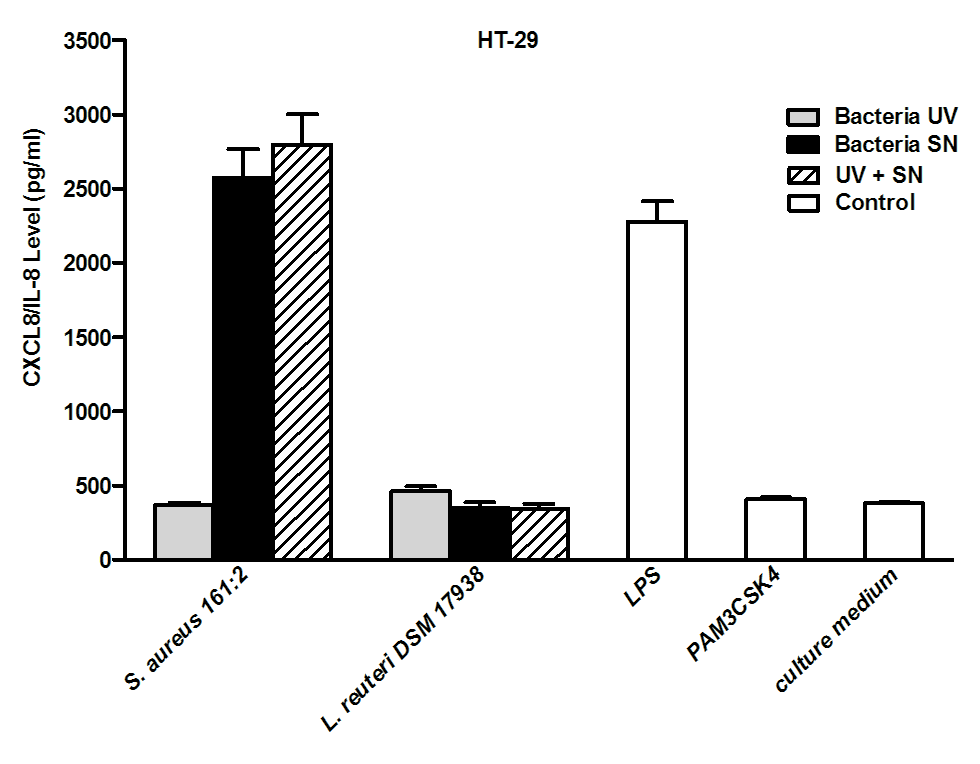

Supplement: Figure S1 — Cytokine production by IEC stimulated with bacteria (supernatant or UV-killed). Level of CXCL8/IL-8 in HT29 supernatant collected after co-cultured with Lactobacillus and Staphylococcus strain-sn and/or Ultraviolet-killed (UV) bacteria, respectively. The graph represent mean + SEM value of 3 independent experiments. Background generated by bacterial medium is subtracted. (TIF) [file pone.0077893.s001.tif]
